# Growing Taller without Hormones? Dr. Consult Google—An Evaluation of Online Information Related to Limb Lengthening

**DOI:** 10.3390/healthcare11020172

**Published:** 2023-01-06

**Authors:** Sefa Key, Mustafa Yalın, Mehmet Erten

**Affiliations:** 1 Department of Orthopedics and Traumatology, Faculty of Medicine, Firat University, Elazığ 23190, Turkey; 2 Department of Orthopedics and Traumatology, Fethi Sekin City Hospital, Elazığ 23280, Turkey; 3 Department of Medical Biochemistry, Malatya Training and Research Hospital, Malatya 44100, Turkey

**Keywords:** limb lengthening, information, websites, surgery, eHealth

## Abstract

Purpose: The aim of this study was to investigate the reliability, content and readability of the information available on the Internet related to limb lengthening surgeries, which have recently been progressively in fashion. Methods: The three most commonly used browsers on the Internet were determined and a search term for “Limb Lengthening Surgery” was typed for each browser. The websites were categorized by their type, and the content and the quality of them was evaluated using the DISCERN score, the Journal of American Medical Association (JAMA) benchmark and the Global Quality Score (GQS). The Flesch Kincaid Grade Level (FKGL) and the Flesch Reading Ease Score (FKRS) were used to evaluate the readability. Each website also assessed the presence (or absence) of the Health on Net (HON) code. Results: The academic category was found to be significantly higher than the medical and commercial categories. Mean FKGL and FCRS scores, DISCERN score values, JAMA, GQS and LLCS score values of Websites with HON code were significantly higher than those without. Conclusions: The quality of online information related to limb lengthening was of low quality. Although some websites, especially academic resources, were of higher quality, the readability of their content is just about 2.5 degrees higher than the sixth-grade reading level.

## 1. Introduction

Limb lengthening is a rapidly developing field of orthopedic surgery that has today become a standard procedure, with its implementation expanding to include the upper extremities and cosmetic lengthening [1]. In recent years, people have increasingly used online data to reach health information and determine treatment preferences [2].

Despite its widespread use by doctors and patients, there are some controversial aspects to this easy accessibility to a huge amount of information, such as concerns regarding the authenticity of the data and industry bias [3]. A study conducted in the United States found that Internet access of the American public increased to 93% by 2021 [4]. Patients are applying to the Internet with rising frequency to search their orthopedic issues and treatment preferences. In spite of the prevalence of patients using the Internet to search their orthopedic issues, the quality of the online data is quite disputable [5].

Limb lengthening could have many aspects. On one hand, deformities caused by biochemical abnormalities are diseases for which medical treatment cannot be effective [6]. Achondroplasia is the most common form of congenital short stature (dwarfism) [7]. Mutations in the fibroblast growth factor receptor type 3 (FGFR3) gene identified individuals with achondroplasia [8]. Currently, short-term growth hormone (GH) therapy is used in patients with achondroplasia. GH treatment is the approved therapy in Japan since 1997 [9]. Moreover, trials of long-term GH therapy have also been reported [9]. In limb lengthening procedures, GH therapy can be applied in combination with surgical treatment or alone [9,10,11]. X-linked hypophosphatemia is another rare one, which causes a dysregulation of the fibroblast-like growth factor 23 (FGF23) [8]. Phosphate and active vitamin D supplementation tried to reduce musculoskeletal symptoms, including musculoskeletal pain/weakness or joint pain, and lower extremity deformity [6,7,12].

To our knowledge, no printed report assessing the online data for limb lengthening surgery was conducted. We therefore conducted a study to determine: the content, quality and readability of the information about limb lengthening available online. We hypothesized that the content and the quality of online information related to limb lengthening would be acceptable and sufficient. Furthermore, we hypothesized that the readability of online data would be comprehensible. This study aimed to fill this research gap by evaluating the content, quality and readability of the information about limb lengthening surgery available online.

## 2. Materials and Methods

**Figure 1:**
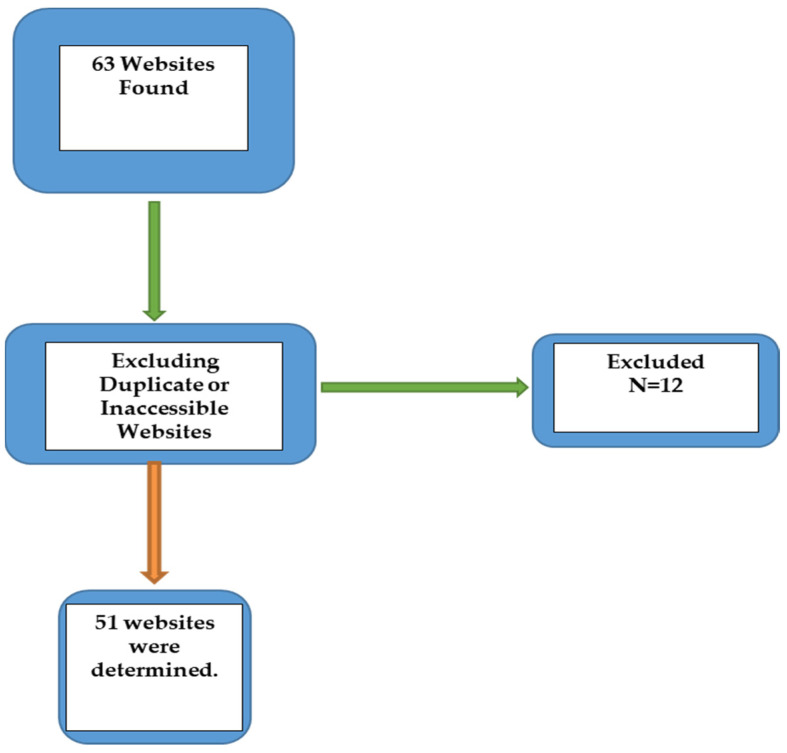
Flowchart of our study.

Scanning was carried out to account for previous studies in this field [1,13,14]. As of August 2022, Google was the predominant search engine, with a 69.80% market share, followed by Bing (13.31%) and Yahoo! (2.11%) [15]. Hence, searches were conducted particularly on these three browsers by using the term ‘Limb Lengthening Surgery’. All searches were carried out on the same day (10 August 2022), and all cookies were removed away from the browsers before the search was initiated. The top 40 websites in each of the browsers were evaluated in the study. After excluding duplicate or inaccessible websites which require payment for access to information, 51 websites were determined (Figure 1). The content and assessment scores of the websites were specified separately by the two authors of this study, who attentively analyzed each website. Subsequently, the websites were categorized as academic, physician, medical and commercial.

### 2.1. Methods of Assessment

All the selected websites were evaluated using the DISCERN instrument [16], the Journal of American Medical Association (JAMA) benchmark, the Global Quality Score (GQS), the Flesch-Kincaid Readability Test Tool (FK), the Limb Lengthening Content Score (LLCS) (Table 1) and the presence or absence of the Health On the Net (HON) Foundation seal.

The DISCERN instrument, which comprises 16 questions that each ranked on a 5-point scale (a score of 1 means the criterion is not met, a score of 2 to 4 means the criterion is relatively met and 5 means the criterion is entirely met) was applied. DISCERN is the most commonly used tool for measuring the quality of health information [17]. Since each question is ranked from 1 to 5, the minimum and maximum total scores for this tool are 6 and 80, respectively. The JAMA benchmark criteria were used to define each website on four likely sections: (1) authorship; (2) listing of references used; (3) disclosure of ownership, sponsorship, funding and (4) the date of update. One point was given for each criterion and a maximum score of 4 points was awarded for this evaluation [18].

The GQS, which consists of a 5-point measurement to rank the comprehensive quality of the websites, was also determined for each website in our sample. The scores ranked the website’s information quality and its possible benefits to the patient [19].

Among the many methods used to test readability, the Flesch-Kincaid Grade Level (FKGL) and the Flesch Reading Ease Score (FKRS) are the most commonly cited [20,21]. The FKGL of a written text identifies that a person with reading abilities equal to that of someone who has graduated from that ‘academic grade’ will be able to read and figure out the given material (5 was the lowest level and 12 was the highest). FKRS test helps determine the level of education required to easily read a given text. Scores close to 100 mean that the document is easy to read, while scores close to zero suggest that the document is quite complex and difficult to understand. Each website’s text was transferred to a Microsoft Word (Redmond, Washington) document to obtain the FK scores, as practiced in previous studies [22,23,24].

Additionally, to determine the actual content of the websites, an LLCS was generated, which included terms that were determined by the two deformity surgeons (Table 1). One point was allocated for the mention of each of the predefined terms related to general aspects of the symptoms, diagnostic tools, treatment options and complications for limb lengthening.

Websites were ranked from 0 to 30, with 30 defining a website with maximal content quality. The LLCS scoring of the websites was independently carried out by the two authors of this study.

Finally, the presence (or absence) of the Health on Net (HON) code was recorded for quality. The HON Foundation is a nonprofit nongovernmental organization that was established in 1996 and established ethical standards for publishing medical information on the Internet. It is the most commonly used online reliability code for medical information [25].

### 2.2. Statistical Analysis

While evaluating the findings obtained in the study, IBM SPSS Statistics 22 (IBM SPSS, Turkey) program was used for statistical analysis. While evaluating the study data, the conformity of the parameters to the normal distribution was evaluated with the Shapiro Wilks test. In addition to descriptive statistical methods (mean, standard deviation, frequency), the Kruskal Wallis test was used for the comparison of the parameters that did not show normal distribution in the comparison of quantitative data, and the Dunn’s test was used to determine the group that caused the difference. The Mann-Whitney U test was applied for the comparison between two groups of parameters that did not show normal dispersion. Spearman’s rho correlation analysis was used to evaluate the relationships between parameters that did not conform to the normal dispersion. Intra-class correlation coefficient, lower and upper limits were calculated to determine the levels of agreement between observers. Significance was evaluated at the *p* < 0.05 level.

## 3. Results

**Figure 2:**
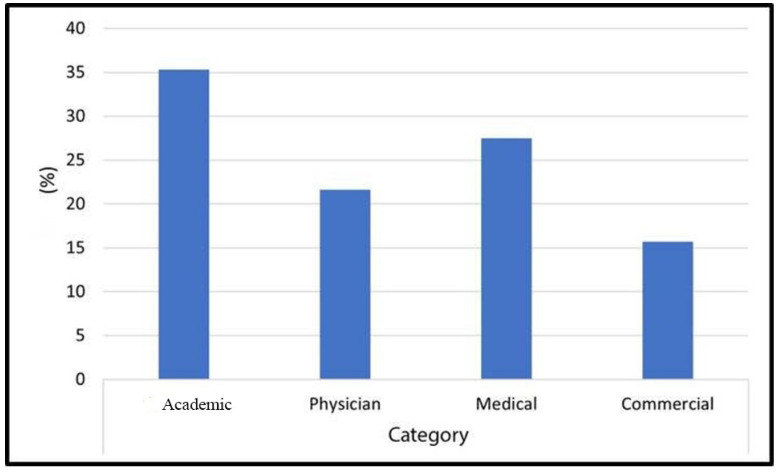
Distribution of websites according to sources.

First, a total of 51 websites were classified according to their sources: 35.3% were academic, 21.6% physician, 27.5% medical and 15.7% commercial (Figure 2).

The average DISCERN score was 33.92 ± 13.28, the mean JAMA benchmark score was 2.06 ± 0.89 out of 4 and also the average FKGL and FCRS scores were 8.43 ± 1.98 and 49.85 ± 14.66, respectively (Table 2).

Statistically significant differences were observed between the categories in terms of their DISCERN scores (*p*: 0.021, *p* < 0.05), JAMA scores (*p*: 0.010, *p* < 0.05), GQS scores (*p*: 0.020, *p* < 0.05) and LLCS scores (*p*: 0.006, *p* < 0.05). In the post-hoc evaluations conducted to determine the categories from which the significance originated, DISCERN JAMA, GQS score and LLCS scores of the academic category were found to be significantly higher than the medical and commercial categories. Moreover, there was no significant difference between the other categories in terms of their DISCERN, JAMA and GQS scores (Table 3).

We found a positive, 83%, and statistically significant correlation between the DISCERN and JAMA scores (*p* < 0.05). A statistically significant correlation was also identified between the DISCERN and LLCS scores at the 62.1% level, and between the JAMA and LLCS scores at the 47.3% level (*p* < 0.05) (Table 3).

Furthermore, while 86.3% of websites did not have a HON code, 13.7% of them did. The DISCERN score values of websites with a HON code were significantly higher than those without it (*p* < 0.05). Apart from this, the JAMA, GQS and LLCS score values of websites with a HON code were found to be significantly higher than those without it (*p* < 0.05). However, there was no statistically significant difference between websites with and without a HON code in terms of their FKGL and FCRS scores (*p* = 0.8) (Table 4).

## 4. Discussion

Technology has revolutionized our lives, with the Internet becoming the most popular origin of data, including information related to health [26]. Several studies have reported that the accuracy and quality of health-related information available on the Internet is quite low [27]. It is important to evaluate online resources and help patients find high-quality and complete content on readable websites, since low-quality information can negatively impact the relationship between patients and doctors, resulting in negative outcomes [27].

The findings in this study, drawn from analyses conducted with standard evaluation tools, demonstrated that websites that were easily accessible to someone seeking information on the topic of limb lengthening were often of low quality. These results were similar to those of previous orthopedic studies on information quality [14,28]. The primary issues that plague Internet-based information are the lack of any control mechanism and issues related to auditing.

People can even create web pages without having sufficient experience and knowledge. This could result in misdirection, especially for a patient seeking information on any health problem. This situation may negatively affect the expectations of patients regarding diagnosis and treatment plans, thus affecting the dynamics of a patient–physician relationship [28].

In the current study, the DISCERN, JAMA, GQS and LLCS scores of the academic category were found to be significantly higher than the other categories. Consistent with previous studies [14,29] the academic and physician categories scored higher in terms of information content and quality in the present study. In contrast, Agar et al. [13] found no significant relationship between groups and their quality scores in their 2022 study. These results indicate that the quality and content of the information available on the Internet are variable, even when it is an academic study.

The average DISCERN score for the sample considered in this study was 33.92 ± 13.28. This result is coherent with prior studies reported in the literature [30,31], which highlights that the quality of information available on websites is low. The reason behind this low average score could be because websites do not efficiently assess the purpose of their content and fail to provide referenced, reliable information in their text.

**Figure 3:**
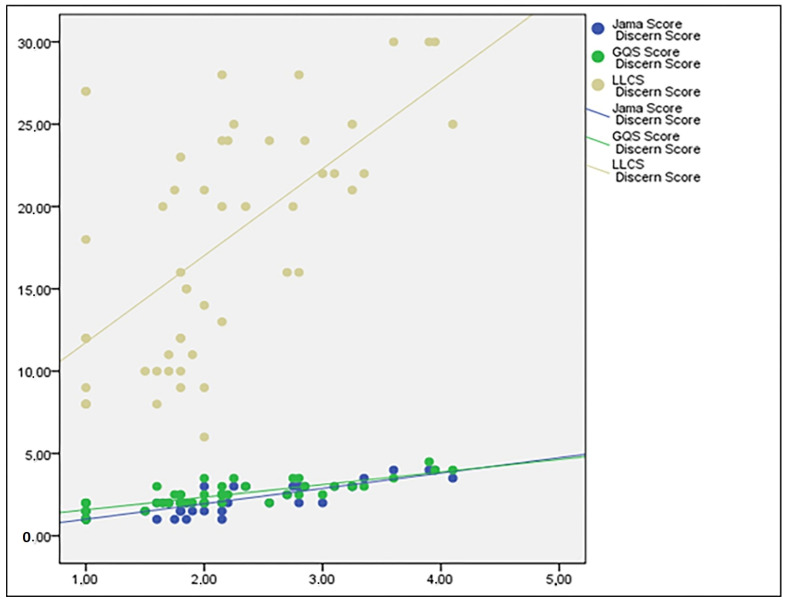
Relationship of DISCERN score to other scores.

The mean JAMA benchmark score was 2.06 ± 0.89 out of 4, much the same as reported in previous studies [27]. The reason for the low JAMA scores could be that most websites did not indicate any references or resources. We noted that the JAMA benchmark criteria had a positive correlation with the DISCERN score (*p* < 0.001) (Figure 3).

**Figure 4:**
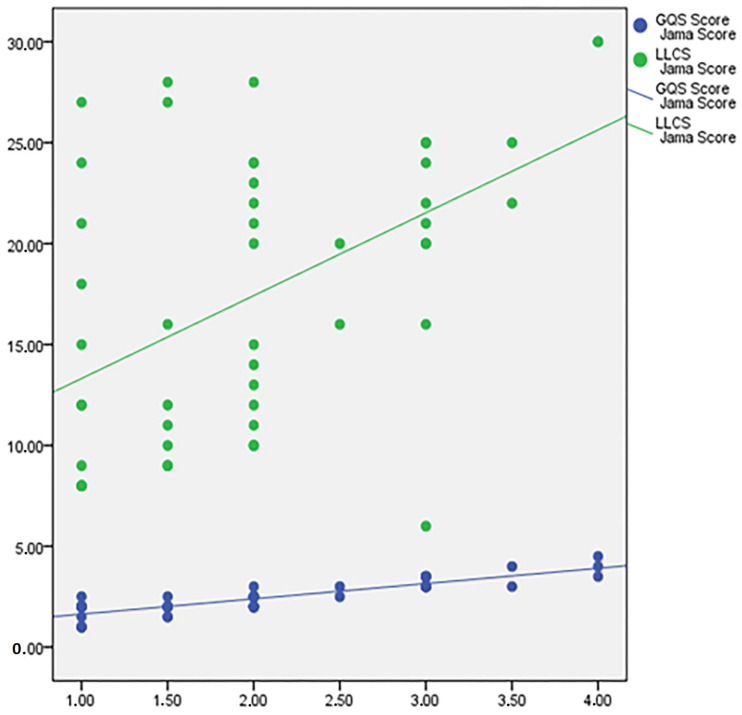
Relationship of JAMA score to other scores.

This might be expounded by the fact that two questions regarding the DISCERN score were concerned with the presence of references and the date of publication, both of which are also significant sections of the JAMA benchmark criteria score. In addition, we identified an 84.9% statistically significant relationship between the JAMA and GQS scores, as well as a positive 47.3% statistically significant relationship between the JAMA and LLCS scores (Figure 4).

The current study demonstrated that the average FKGL and FCRS scores were 8.43 ± 1.98 and 49.85 ± 14.66, respectively. According to these results, the FKGL score is just about 2.5 degrees higher than the sixth grade reading level suggested by the American Medical Association (AMA) and the National Institutes of Health (NIH) [32]. That is, to understand the information presented, it is necessary to have at least a 6th grade English language level, but it should be taken into account that there may be differences in language and personal characteristics. This result is consistent with the results of other studies that have assessed the readability of online information [33,34]. The FCRS score obtained in this study indicates that the online information was “hard to read”, signifying that patients must have almost a high school level qualification in English to understand the content of the online information appropriately.

As priorly reported [5,35], the quality of online publications with a HON code was higher, advocating that the content of websites with a HON code can be trusted to provide higher quality and more accurate information. In the current study, 86.3% of websites did not have a HON code, while 13.7% of them did. The DISCERN, JAMA, GQS and LLCS score values of the content evaluated with regard to websites with a HON code were found to be significantly higher than those without a HON code (*p* < 0.05). However, no statistically significant difference was identified between websites with and without HON codes in terms of their FKGL and FCRS scores.

### Limitations

First, since the content score used in this study was created from information provided by two orthopedic deformity surgeons, it may not be sufficiently comprehensive. Second, our study focused on analyzing only online printed materials, but patients may also use audio-visual material to obtain information, and this has not been assessed in the present study. Search results or ranking orders may change frequently because the Internet is constantly evolving. Although we deleted cookies, search results can vary from person to person at any time. Furthermore, this study did not assess the quality of information on websites other than the three most frequently used search engines. To our knowledge, this study is the first of its kind related to limb lengthening. In this respect, the current study can help to evaluate information that may be significant for maintaining equilibrium in patient–doctor connections. On the other hand, rare diseases such as Osteogenesis imperfecta, congenital pseudarthrosis of the tibia, etc., cause limb deformities. Rare diseases are also crucial for understanding the digital age because all patients do not have the same chance to reach appropriate health aid. Nevertheless, most of us have internet connections, which is an incredible opportunity that no human being has ever had before. In addition, one must keep in mind that there is an exception for the digital dilemma.

## 5. Conclusions

Consistent with previous research, most of the information on the websites evaluated in this study was of low quality. Although some websites, especially academic resources, are of higher quality, the readability of their content was hard to understand. This makes it difficult for patients to figure out the information they seek concerning limb lengthening. Auditable information source websites that can provide accurate and effective information can be targeted at the general readership. For this purpose, language features, usage area, and outlines of the local features should be targeted to the widest audience. More studies on this subject may contribute to this purpose. As the topic of limb lengthening is an increasingly popular topic, it is necessary for specialists to create websites, which contain accurate information that patients can understand, and directs patients correctly.

## Figures and Tables

**Table 1:** Limb Lengthening Content Score (LLCS).

Limb lengthening
Upper Extremity Limb Lengthening
Achondroplasia
Deformity
Limb-Length Discrepancy
Soft Tissue Coverage
Angular Deformities
Bone Quality
Motion
Stability
Osteotomy
Maturity
Treatment
Complication
Pseudoarthrosis
X-Ray
Decision
Computer Analysis
Technology
Distraction Osteogenesis
Weight Bearing
Ilizarov
External Fixator
Intramedullary Nail
Bone Healing
Special Surgery
Indications
Reconstructive Surgery
Rehabilitation
External/Internal Implant

**Table 2:** Minimum, maximum, mean, standard deviation and median values of scores.

Title	Min–Max	Mean ± SD	Median
**Discern reviewer 1**	16–64	33.44 ± 12.96	30.4
**Discern reviewer 2**	16–67.2	34.4 ± 13.92	32
**Discern score**	16–65.6	33.92 ± 13.28	32
**Jama reviewer 1**	1–4	2.06 ± 0.93	2
**Jama reviewer 2**	1–4	2.06 ± 0.93	2
**Jama Score**	1–4	2.06 ± 0.89	2
**GQS reviewer 1**	1–4	2.39 ± 0.7	2
**GQS reviewer 2**	1–5	2.49 ± 0.95	2
**GQS Score**	1–4.5	2.44 ± 0.77	2.5
**FKGL**	5–11.9	8.43 ± 1.98	8.5
**FKRS**	21.2–94.9	49.85 ± 14.66	48.5
**LLCS**	6–30	17.67 ± 7.14	18

**Table 3:** Evaluation categorical scores.

Category	Discern Score	JAMA Score	GQS	FKGL	FKRS	LLCS
	Mean ± SD (Median)	Mean ± SD (Median)	Mean ± SD (Median)	Mean ± SD (Median)	Mean ± SD (Median)	Mean ± SD (Median)
**Academical**	40.64 ± 14.88 (38.4)	2.61 ± 0.93 (2.8)	2.86 ± 0.92 (3)	8.92 ± 1.75 (8.8)	45.07 ± 12.38 (44.2)	21.11 ± 7.07 (23)
**Physician**	33.92 ± 11.36 (30.4)	1.95 ± 0.91 (2)	2.45 ± 0.52 (2.5)	8.16 ± 2.3 (7.9)	52.74 ± 20.01 (55.3)	20.09 ± 5.15 (21)
**Medical**	29.92 ± 10.24 (28.8)	1.75 ± 0.67 (1.5)	2.21 ± 0.58 (2)	8.12 ± 1.98 (8.1)	52.09 ± 11.17 (50.4)	13.79 ± 5.79 (12)
**Commercial**	25.92 ± 11.04 (25.6)	1.5 ± 0.46 (1.5)	1.88 ± 0.52 (2)	8.25 ± 2.17 (8.3)	52.68 ± 16.41 (49.6)	13.38 ± 7.21 (10.5)
*p*	0.021 *	0.010 *	0.020 *	0.599	0.290	0.006 *

Kruskal Wallis Test, * *p* < 0.05.

**Table 4:** Evaluation of scores according to the presence of HON code.

Score	HON		
	Absent	Present	*p*
	Mean ± SD (Median)	Mean ± SD (Median)	
**DISCERN Score**	30.88 ± 11.04 (28.8)	52.96 ± 10.88 (54.4)	0.000 *
**JAMA Score**	1.86 ± 0.76 (2)	3.29 ± 0.7 (3.5)	0.000 *
**GQS Score**	2.3 ± 0.7 (2)	3.36 ± 0.56 (3.5)	0.001 *
**FKGL**	8.4 ± 2.05 (8.5)	8.61 ± 1.57 (8.4)	**0.913**
**FKRS**	50.72 ± 15.04 (50)	44.36 ± 11.4 (41.6)	**0.129**
**LLCS**	16.73 ± 6.91 (16)	23.57 ± 5.97 (25)	0.018 *

Mann Whitney U Test, * *p* < 0.05.

## Data Availability

The authors prefer not to share study data.

## Abbreviations

American Medical Association	AMA
Fibroblast Growth Factor Receptor type 3	FGFR3
Fibroblast-like Growth Factor 23	FGF23
Flesch Kincaid Grade Level	FKGL
Flesch Reading Ease Score	FKRS
Flesch-Kincaid Readability Test Tool	FK
Global Quality Score	GQS
Growth Hormone	GH
Health on Net	HON
Journal of American Medical Association	JAMA
Limb Lengthening Content Score	LLCS
National Institutes of Health	NIH
